# Reply to Marino et al. Does Subretinal Fluid Influence Choroidal Thickness (ChT) and Structure in Preeclampsia with Serous Retinal Detachment? Comment on “Fukui et al. Changes in Choroidal Thickness and Structure in Preeclampsia with Serous Retinal Detachment. *J. Clin. Med.* 2023, *12*, 609”

**DOI:** 10.3390/jcm12093274

**Published:** 2023-05-04

**Authors:** Ayumi Fukui, Hiroshi Tanaka, Nobuhiro Terao, Kenji Nagata, Akifumi Matsumoto, Natsuki Kusada, Kentaro Kojima, Chie Sotozono

**Affiliations:** Department of Ophthalmology, Kyoto Prefectural University of Medicine, 465 Kajii-cho, Hirokoji-agaru, Kawaramachi-dori, Kamigyo-ku, Kyoto 602-0841, Japan

We thank the authors for the interesting comments [1] on our paper [2], and for truly appreciating our work as well.

As you pointed out, axial length and choroidal thickness are known to be thicker in the shorter eyes and thinner in the longer eyes [3]. In the case of the present study, the axial length should have been corrected for SCT measurement. However, since this is a retrospective study, and the number of cases in which axial length was measured was small, the results were not limited to cases in which axial length was measured. This is also because only the minimum necessary examination was performed to shorten the examination time for patients whose general condition was not stable, such as pregnant women who were about to give birth, patients who had just undergone cesarean sections, and patients suffering from pregnancy complications. In addition, we would like to add that the refractive error, in this case, was normal to moderate myopia, and we assume that cases with extremely short or long eyes were not included in the examination. When we examine similar cases in the future, we will also measure the axial length, although it will depend on the patient’s general condition.

Regarding your second point, as you pointed out, it is difficult to identify how to locate the macular fovea when SRD is present. The current study was retrospective and used the method of measuring SCT from previously scanned OCT images. The location of the central fovea was confirmed from the images with OCT when SRD improved, and the location of the fovea on OCT when SRD was present was estimated using the site of the choroidal vessels. In the future, we would like to use the follow-up setting as you indicated.

CVI is a standardized, newly defined parameter recently attracting attention because it allows quantitative evaluation of choroidal vessels. However, it has been reported that accurate CVI measurement is difficult due to retinal vascular shadows affecting the choroid, corneal opacity, and poor fixation [4], so it may not yet be a standardized parameter. This study used Niblack’s auto-local threshold technique to measure CVI based on previous reports [5,6]. Niblack’s auto local threshold technique is a binarization method that considers the mean value and standard deviation of all pixels in the selected region and was adopted because it is considered to be relatively less affected by signal amplification.

## Data Availability

The data in this study is available upon a reasonable request to the corresponding author, as it is not publicly available due to privacy reasons and ethical concerns.
